# Circulating circRNA as biomarkers for dilated cardiomyopathy etiology

**DOI:** 10.1007/s00109-021-02119-6

**Published:** 2021-09-08

**Authors:** Marina C. Costa, Maria Calderon-Dominguez, Alipio Mangas, Oscar Campuzano, Georgia Sarquella-Brugada, Mónica Ramos, Maribel Quezada-Feijoo, José Manuel García Pinilla, Ainhoa Robles-Mezcua, Galan del Aguila Pacheco-Cruz, Thalia Belmonte, Francisco J. Enguita, Rocío Toro

**Affiliations:** 1 Faculdade de Medicina, Instituto de Medicina Molecular João Lobo Antunes, Universidade de Lisboa, Av. Prof. Egas Moniz, 1649-028 Lisbon, Portugal; 2 Biomedical Research and Innovation Institute of Cadiz (INiBICA), Research Unit, Puerta del Mar University Hospital, Cadiz, Spain; 3 Internal Medicine Department, Puerta del Mar University Hospital, Cadiz, Spain; 4 Medicine Department, School of Medicine, University of Cádiz, Cadiz, Spain; 5 Medical Science Department, School of Medicine, University of Girona, Girona, Spain; 6 Cardiovascular Genetics Center, University of Girona-IDIBGI, Girona, Spain; 7 Centro de Investigación Biomédica en Red, Enfermedades Cardiovasculares (CIBERCV), Madrid, Spain; 8 Cardiology Department Hospital Cruz Roja, Alfonso X University, Madrid, Spain; 9 Servicio de Cardiología, Unidad de Insuficiencia Cardíaca Y Cardiopatías FamiliaresHospital Universitario Virgen de La VictoriaIBIMA, Malaga, Spain; 10 Consumo Y Bienestar Social, CIBER-Cardiovascular, Instituto de Salud Carlos III, Ministerio de Sanidad, Madrid, Spain

**Keywords:** Circulating circular RNA, Ischemic-dilated cardiomyopathy, Lamin A/C-dilated cardiomyopathy

## Abstract

**Abstract:**

Dilated cardiomyopathy (DCM) is the third most common cause of heart failure. The multidisciplinary nature of testing — involving genetics, imaging, or cardiovascular techniques — makes its diagnosis challenging. Novel and reliable biomarkers are needed for early identification and tailored personalized management. Peripheral circular RNAs (circRNAs), a leading research topic, remain mostly unexplored in DCM. We aimed to assess whether peripheral circRNAs are expressed differentially among etiology-based DCM. The study was based on a case–control multicentric study. We enrolled 130 subjects: healthy controls (*n* = 20), idiopathic DCM (*n* = 30), ischemic DCM (*n* = 20), and familial DCM patients which included pathogen variants of (i) *LMNA* gene (*n* = 30) and (ii) BCL2-associated athanogene 3 (*BAG3*) gene (*n* = 30). Differentially expressed circRNAs were analyzed in plasma samples by quantitative RT-PCR and correlated to relevant systolic and diastolic parameters. The pathophysiological implications were explored through bioinformatics tools. Four circRNAs were overexpressed compared to controls: hsa_circ_0003258, hsa_circ_0051238, and hsa_circ_0051239 in *LMNA*-related DCM and hsa_circ_0089762 in the ischemic DCM cohort. The obtained areas under the curve confirm the discriminative capacity of circRNAs. The circRNAs correlated with some diastolic and systolic echocardiographic parameters with notable diagnostic potential in DCM. Circulating circRNAs may be helpful for the etiology-based diagnosis of DCM as a non-invasive biomarker.

**Key messages:**

- The limitations of cardiac diagnostic imaging and the absence of a robust biomarker reveal the need for a diagnostic tool for dilated cardiomyopathy (DCM).
- The circular RNA (circRNA) expression pattern is paramount for categorizing the DCM etiologies.
- Our peripheral circRNAs fingerprint discriminates between various among etiology-based DCM and correlates with some echocardiographic parameters.
- We provide a potential non-invasive biomarker for the etiology-based diagnosis of LMNA-related DCM and ischemic DCM.

**Supplementary Information:**

The online version contains supplementary material available at 10.1007/s00109-021-02119-6.

## Introduction

Heart failure is a global pandemic affecting more than 25 million people worldwide, with a continuously increasing prevalence [1]. One of the major causes of heart failure is dilated cardiomyopathy (DCM), characterized by chamber enlargement and contractile dysfunction of the left ventricle (LV) [2]. Several etiologies are included in the DCM common pathway. Ischemic cardiomyopathy is more common than non-ischemic (59% compared with 41%) [2]. Among non-ischemic cardiomyopathy, up to 35% of idiopathic DCM may have a family history [2, 3]. Pathogenic alterations in the gene encoding nuclear lamin A and C proteins-lamin A/C (*LMNA*) explain 5–10% of familial DCM cases.

DCM is a heterogeneous entity that has different outcomes and may require diverse therapies [4]. Notably, ischemic and familial DCM are major groups with life-threatening arrhythmias [3]. *LMNA*-related DCM presents highly aggressive outcomes and lethal ventricular arrhythmias [5]. Male sex, LV ejection fraction (LVEF) lower than 50%, and non-missense mutations are independent predictors of adverse outcome [6]. Thus, the identification of DCM etiology may help clinicians to stratify patients at risk of fatal events. However, the diagnosis process to reach DCM etiology involves several clinical steps. Multidisciplinary teams, imaging tests, the high cost of genetic testing, and its low efficiency make DCM etiologic diagnosis challenging. A precise, accessible biomarker that supports this process is required to improve diagnosis and early identification of asymptomatic cases. This would facilitate the adoption of tailored management.

Non-coding RNAs have pivotal roles in regulating the network that governs the physiology and pathology of cardiovascular diseases [7]. To date, microRNAs (miRNAs) have been considered a more relevant biomarker candidate due to the complexity of circular RNA (circRNA) assessment in human screening [8]. However, circRNAs also have thought-provoking features. The advantages of circRNAs are their cell type, tissue, and developmental stage specificity. Furthermore, they are independently regulated and more stable than lineal RNA, and they are gathered in cells and human body fluids [8]. CircRNAs modulate gene expression by sponging miRNAs, interacting with RNA-binding proteins (RBPs), and competing with canonical splicing of their pre-mRNA precursor [9]. Research reporting circRNAs as an effective diagnostic and therapeutic biomarker in many diseases has grown exponentially in the last decade. Nevertheless, the potential of using this easy-to-monitor and highly stable marker for stratifying DCM etiologies remains unexplored. Additionally, the experimental and computational analyses of these molecular cross-regulations will propel new insights on DCM [8].

The present study aimed to identify differentially expressed circRNAs in the plasma of patients with DCM of various etiologies such as familial, idiopathic, or ischemic.

## Material and methods

### Study design

**Fig. 1:**
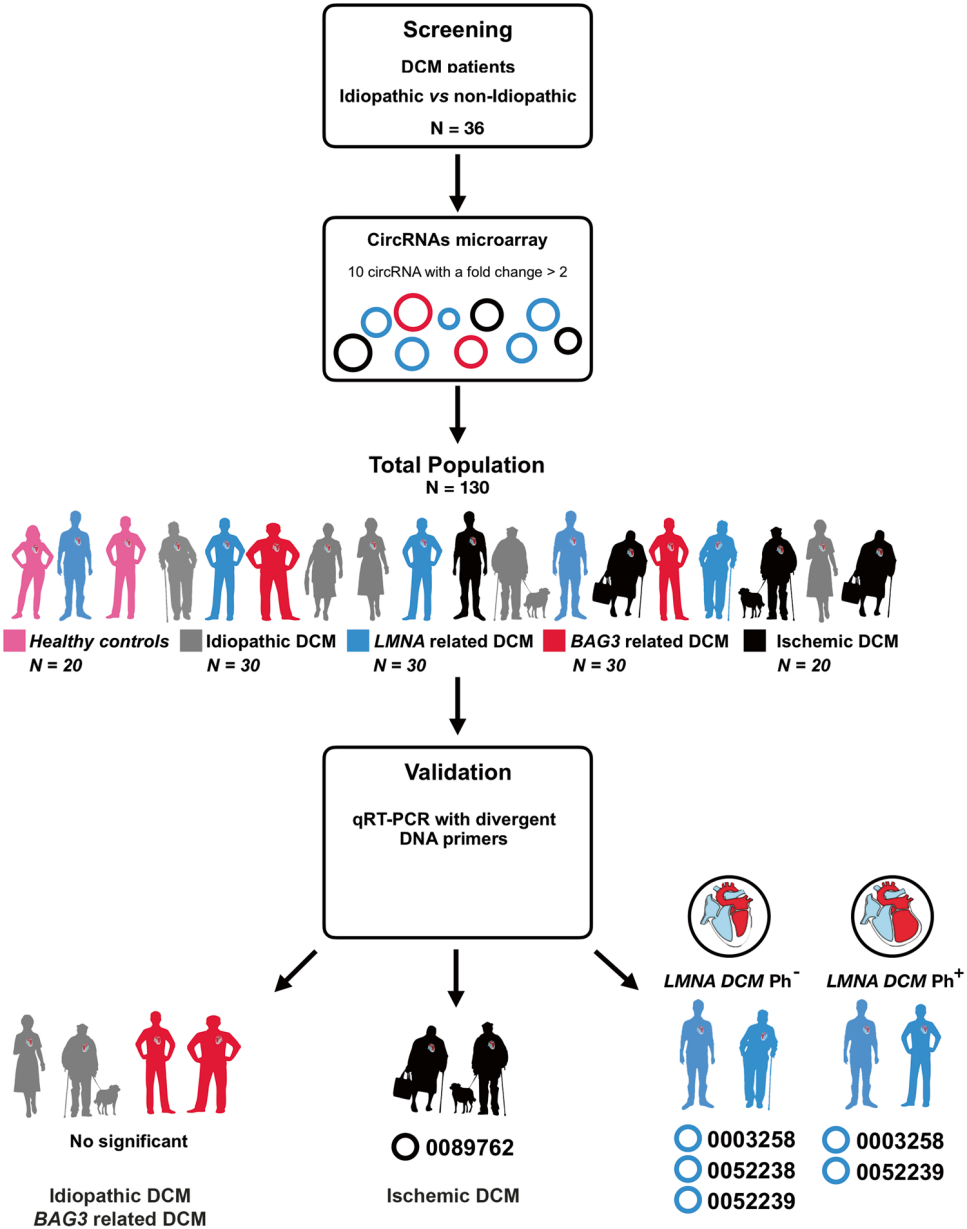
Flowchart of the study design strategy. This figure illustrates the experimental workflow of the study including screening, validation, and peripheral circRNAs overexpressed for the *LMNA*^Ph−^, *LMNA*^Ph+^, and ischemic DCM cohort. Abbreviations: *BAG3,* BCL2-associated athanogene 3; DCM, dilated cardiomyopathy; lamin A/C; *LMNA*^Ph*−*^, *LMNA* carrier of the pathogenic variant; *LMNA*^Ph+^, *LMNA* carrier phenotype positive; LVEF, left ventricle ejection fraction

The study was based on a case–control multicentric study**.** Patient samples and the dataset were collected from several centers (Puerta del Mar University Hospital, Cádiz; Cruz Roja Hospital, Madrid; and Virgen de la Victoria University Hospital, Málaga, Spain). We enrolled 130 subjects distributed in five study groups: healthy controls (*n* = 20), idiopathic DCM (*n* = 30), ischemic DCM (*n* = 20), and familial DCM patients. The carriers of rare pathogenic variants included were (i) *LMNA* gene (*n* = 30) and (ii) *BCL2-associated athanogene 3* (*BAG*3) gene (*n* = 30) (Fig. 1).

DCM etiology was determined by three independent clinical cardiologists, who are experts in cardiomyopathies. DCM was defined as either LVEF levels below 50% and/or LV end-diastolic diameter larger than 56 mm [10]. *BAG3* and *LMNA* participants were confirmed genetically and fulfilled the diagnostic clinical criteria for familial DCM [11]. The *LMNA* cohort was subclassified as a carrier of the pathogenic variant, phenotypically negative (*LMNA*^*Ph−*^) and genetically and phenotypically positive (*LMNA*^Ph+^) as previously described [11]. Genetic etiology was ruled out in all idiopathic DCM patients. Ischemic DCM was diagnosed if a precedent of acute myocardial infarction or coronary artery disease was shown, which developed LV remodeling and dysfunction [10]. A transthoracic echocardiography protocol was performed as described previously [11, 12]. The information included anthropometric, clinical, therapeutic, electrocardiographic, and echocardiographic data from electronic medical records (Table 1).

**Table 1:** Study population: anthropometric, clinical, and echocardiographic variables

Variable	Healthy control (*N* = 20)	Idiopathic (*N* = 30)	*LMNA*^Ph−^ (*N* = 12)	*LMNA*^Ph+^ (*N* = 18)	*BAG*3 (*N* = 30)	Ischemic (*N* = 20)
Age (years)	42.0 ± 11.0	63.7 ± 8.2	40.6 ± 6.9	38.7 ± 15.0	42.2 ± 14.8	71.1 ± 8.5
Sex (male)	55%	70%	23.1%	42.9%	68.4%	72.2%
BMI (kg/m^2)^	25.1 ± 3.3	26.7 ± 2.6	25.4 ± 2.1	23.6 ± 3.9	27.9 ± 4.9	28.8 ± 4.9
Heart rate (bpm)	65.7 ± 11.9	71 ± 13.9	65.7 ± 5.9	64.3 ± 9.9	73 ± 10	64.6 ± 16.8
Smoker	0%	60%	57.1%	30.8%	26.3%	22.2%
SBP (mm Hg)	114.5 ± 8.7	113.1 ± 11.9	128.4 ± 15.9	123.2 ± 20.9	128.1 ± 13.3	124.3 ± 12.7
DBP (mm Hg)	73.5 ± 8.5	73.1 ± 7.1	81.8 ± 6.1	76.7 ± 17.9	81.1 ± 7.8	72.2 ± 8.6
LVEF (%)	68.8 ± 6.0	30.5 ± 10.2	44.5 ± 5.0	61.0 ± 5.9	49.5 ± 11.9	34.7 ± 7.5
LVEDD (mm)	47.7 ± 4.8	63.0 ± 3.8	58.0 ± 3.4	49.2 ± 12.6	55.6 ± 7.5	58.6 ± 4.8
LVESD (mm)	30.0 ± 6.9	48.1 ± 16.8	43.8 ± 3.1	30.7 ± 6.8	40.4 ± 9.3	44.1 ± 13.2
LA volume (mL/m^2^)	17.4 ± 4.3	71.1 ± 25.0	49.3 ± 12.4	41.0 ± 15.5	68.2 ± 25.8	62.1 ± 19.6
LAD (mm)	35.1 ± 5.4	45.2 ± 9.1	40.8 ± 4.3	33.8 ± 6.6	37.6 ± 6.5	40.8 ± 6.1
RV (mm)	28.6 ± 3.5	39.7 ± 6.5	31.7 ± 1.9	28.8 ± 5.2	32.1 ± 7.6	31.4 ± 6.9
TAPSE	22.2 ± 2.7	18.2 ± 6.4	21.6 ± 3.6	21.3 ± 3.5	21.1 ± 5.4	18.8 ± 3.9
MAPSE	18.1 ± 1.6	9.6 ± 2.7	12.1 ± 3.1	16.0 ± 2.6	12.3 ± 3.2	10.6 ± 2.1
E (cm/s)	0.7 ± 0.2	0.7 ± 0.2	0.8 ± 0.1	0.8 ± 0.2	0.8 ± 0.3	0.8 ± 0.2
A (cm/s)	0.6 ± 0.1	0.8 ± 0.3	0.7 ± 0.3	0.5 ± 0.2	0.6 ± 0.2	0.8 ± 0.3
S’sTDI (cm/s)	0.08 ± 0.01	0.06 ± 0.06	0.06 ± 0.01	0.08 ± 0.02	0.08 ± 0.01	0.05 ± 0.01
E’s TDI (cm/s)	0.09 ± 0.03	0.05 ± 0.05	0.07 ± 0.02	0.10 ± 0.04	0.09 ± 0.04	0.05 ± 0.01
A’s TDI (cm/s)	0.10 ± 0.03	0.06 ± 0.02	0.11 ± 0.02	0.09 ± 0.04	0.11 ± 0.03	0.07 ± 0.03
E/E’ ratio	7.7 ± 2.1	16.3 ± 8.5	10.2 ± 3.0	7.9 ± 2.2	6.3 ± 1.5	15.0 ± 6.4
NYHA functional class (II-III)	0%	10%	14.3%	15.4%	10.5%	11.1%

All values are expressed as mean ± SEM

*A* atrial systolic transmitral flow wave, *A’s TDI* atrial septal mitral annular velocity, *BAG3* BCL2-associated athanogene 3, *BMI* body mass index, *DBP* diastolic blood pressure, *DCM* dilated cardiomyopathy, *E* early diastolic transmitral flow wave, *E′* early diastolic mitral annular velocity, *LA* left atrial, *LAD* left atrial dimension, *LMNA lamin* A/C, *LMNA*^*Ph−*^* LMNA* carrier of the pathogenic variant, *LMNA*^*Ph*+^ *LMNA* carrier phenotypically positive, *LVEDD* left ventricular end-diastolic dimension, *LVEF* left ventricle ejection fraction, *LVESD* left ventricle end-systolic dimension, *MAPSE* mitral annular plane systolic excursion, *NYHA* New York Heart Association classification, *RV* right ventricle, *S*’ positive systolic wave, *SBP* systolic blood pressure, *TAPSE* tricuspid annular plane systolic excursion, *TDI* tissue Doppler imaging

### Ethics

The study protocol was approved by the Andalusian Biomedical Research Ethics committee. The study was performed in full compliance with the Declaration of Helsinki. All participants provided written informed consent.

### Genetic analysis

Genetic analysis was performed as previously described [11]. DNA isolation was undertaken using Chemagic MSM I from whole blood (Chemagic Human Blood). DNA integrity was assessed on 0.8% agarose gel and the quality ratios of absorbance were accomplished using spectrophotometric measurements. dsDNA concentration was determined using fluorometry integrity (Qubit, Life Technologies) and corroborated on 0.8% agarose gel.

### Blood collection

Ten milliliters of peripheral blood was collected in K2-ethylenediaminetetraacetic acid tubes (BD) after 10 h overnight fasting. None of the patients was under heparin therapy. The blood was processed within 4 h after isolation, centrifuged (1500* g*, 15 min, 4 °C), and the plasma layer was aliquoted and stored at − 80ºC until further analysis.

### Microarray analysis

A screening study was carried out using the Arraystar Human Circular RNA Microarray V2.0 (Arraystar, Inc.). This platform analyzed 36 samples of idiopathic and non-idiopathic DCM subjects. Total RNAs from each sample were obtained using the Arraystar’s standard protocols (Arraystar, Inc.). The enriched circRNAs were amplified and transcribed into fluorescent cDNA using a random priming method (Arraystar Super RNA Labeling Kit; Arraystar). The labelled cDNAs were hybridized onto the Arraystar Human circRNA Array V2.0 (Arraystar, Inc.). Once the slides had been washed, they were scanned by the Agilent Scanner G2505C.

### RNA isolation and quantitative reverse transcriptase-polymerase chain reaction

Total RNA was isolated from 200 µL of plasma using a miRNeasy Serum/Plasma Kit (Qiagen). RNA was eluted with 20 µL of RNase-free H_2_O and stored at − 80 °C. For the circRNA quantification, circulating RNA preparations were reverse transcribed with a first-strand cDNA synthesis kit (Nzytech, Portugal) using a random primer approach and following the manufacturer’s instructions. Previous to reverse transcription, samples were spiked with MS2 RNA (Sigma-Aldrich, Germany), which was used as an internal normalizer. Quantification of selected circRNAs was performed by qRT-PCR using divergent DNA primers designed with the circInteractome algorithm [13] (see Supplemental Table 1 for primer sequences) in an Applied Biosystems by the qRT-PCR system. Fold-change analysis between sample groups was calculated by the Delta-Ct method.

### Functional enrichment

Information about circRNAs is available on the circBase website (http://www.circbase.org/). The Circular RNA Interactome (https://circinteractome.nia.nih.gov/) was used to predict miRNAs and RBP-binding sites. The regulatory network was performed with Navigator software [14]. The set of RBPs common to all the differentially expressed circRNAs was analyzed with STRING: functional protein association networks (https://string-db.org) [15]. The set of miRNAs common to all the differentially expressed circRNAs was analyzed with miRNet 2.0 (https://www.mirnet.ca/miRNet/home.xhtml).

### Statistical analysis

Continuous variables are expressed as the mean ± standard deviation. Categorical variables are expressed in frequency and percentage (%). Analysis of variance was applied to compare intergroup circRNAs levels. The Pearson correlation was used to test the link between echocardiographic and clinical variables vs. log_2_ circRNAs. In addition, the association between circRNAs and echocardiography parameters was assessed using logistic bivariate regression. Several models were constructed using the Wilcoxon test and iterating combinations between our circRNA candidates, as well as echocardiographic and clinical covariates. The changes in *p*-values of their variables were evaluated by the Wald test and a likelihood ratio. To characterize the diagnostic performance of the circRNAs candidate, ROC curves were applied together with a logistic regression model to determine the AUC and the specificity and sensitivity of the optimal cutoffs. ROC curves were generated by plotting sensitivity against 100-specificity. Data were presented as the AUC and 95% CI. The statistical software package R (www.r-project.org) was used for all analyses.

## Results

### Analysis of circRNA expression profiles in plasma of DCM patients

A total of 36 idiopathic and non-idiopathic DCM age-matched patients were assessed to test the differences in circRNA expression profiles (see Supplementary Figs. 1 and 2). A total of ten candidate circRNAs (see Supplementary Table 2) were obtained from circRNA microarray screening of plasmatic samples (fold change > 2, *p* < 0.05).

### Validating the expression of the candidate circRNAs

**Fig. 2:**
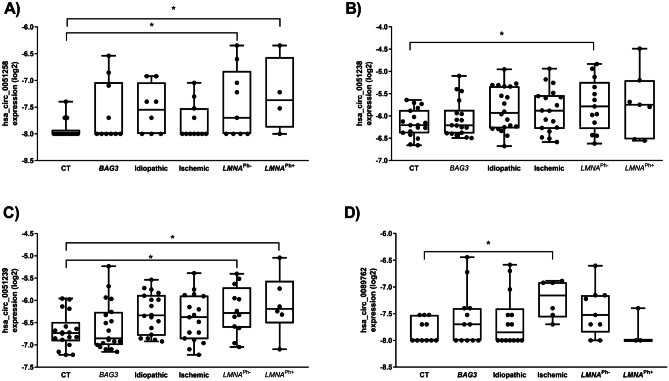
Boxplots of circRNA expression levels, normalized to MS2 RNA, in healthy subjects, *BAG3*-related DCM, idiopathic DCM, ischemic DCM, and *LMNA*-related DCM. The analysis was carried out using qRT-PCR. Data are present in log_2_. Data represent the mean ± SEM. *p < 0.05. Abbreviations: *BAG3,* BCL2-associated athanogene 3; CT, healthy cohort; circRNA, circular RNA; DCM, dilated cardiomyopathy; *LMNA*, lamin A/C; *LMNA*^Ph*−*^, *LMNA* carrier of the pathogenic variant; *LMNA*^Ph+^, *LMNA* carrier phenotype positive

The expression of the ten circRNA candidates was carried out in plasma samples of each study group, using qRT-PCR. Only *LMNA* and ischemic DCM populations showed differential circRNA expression (Table 2). Circulating levels of hsa_circ_0051238, hsa_circ_0051239, and hsa_circ_0003258 were highly upregulated in the *LMNA* population compared to healthy controls (Fig. 2). To assess the strength of circRNAs as an early biomarker before the clinical manifestation of malignant ventricular arrhythmias and LV dilation, the *LMNA*-related DCM group was subdivided into *LMNA* pathogenic variant carrier, phenotypically negative (*LMNA*^Ph−^) and phenotypically positive (*LMNA*^Ph+^). Circulating hsa_circ_0003258 levels were differentially expressed in the *LMNA*^Ph−^ (*LMNA*^Ph−^ *p* = 0.03, *LMNA*^Ph+^ *p* = 0.03) (Fig. 2A). The hsa_circ_0051238 levels were differentially expressed in the *LMNA*^Ph−^ population (*p* = 0.03) (Fig. 2B). And, the hsa_circ_0051239 plasmatic levels were significantly higher in both *LMNA* groups (*LMNA*^Ph−^ *p* = 0.03, *LMNA*^Ph+^ *p* = 0.04) than in healthy subjects (Fig. 2C). Regarding the ischemic DCM cohort, the plasma hsa_circ_0089762 levels were significantly higher (*p* = 0.04) than in healthy subjects (Fig. 2D).

**Table 2:** Peripheral circRNA levels in the study groups

circRNA	CT			*BAG3* DCM				Idiopathic DCM				Ischemic DCM				*LMNA*^Ph−^ DCM				*LMNA*^Ph+^ DCM			
	Med	Q1	Q3	Med	Q1	Q3	*p*	Med	Q1	Q3	*p*	Med	Q1	Q3	*p*	Med	Q1	Q3	*p*	Med	Q1	Q3	*p*
hsa_circ_0003258	− 8	− 8	− 7.92	− 8	− 8	− 7.03	0.45	− 7.55	− 8	− 7.04	0.29	− 8	− 8	− 7.52	0.94	− 7.69	− 8	− 6.82	0.03	− 7.37	− 7.88	− 6.57	0.03
hsa_circ_0051238	− 6.20	− 6.39	− 5.9	− 6.21	− 6.39	− 5.94	0.98	− 5.90	− 6.25	− 5.33	0.10	− 5.87	− 6.30	− 5.51	0.18	− 5.71	− 6.10	− 5.18	0.08	− 5.71	− 5.98	− 5.02	0.03
hsa_circ_0051239	− 6.73	− 6.92	− 6.48	− 6.85	− 7	− 6.26	0.96	− 6.33	− 6.79	− 5.88	0.09	− 6.33	− 6.87	− 5.89	0.34	− 6.28	− 6.61	− 5.71	0.03	− 6.19	− 6.51	− 5.56	0.04
hsa_circ_0089762	− 8	− 8	− 7.52	− 7.69	− 8	− 7.39	0.58	− 7.84	− 8	− 7.40	0.75	− 7.15	− 7.57	− 6.91	0.04	− 7.52	− 7.85	− 7.15	0.25	− 8	− 8	− 7.39	> 0.9

Data presented as median (Q1–Q3). Coefficient significant at *p* < 0.05

*BAG3* BCL2-associated athanogene 3, *CT* healthy control, *DCM* dilated cardiomyopathy, *LMNA* lamin A/C, *LMNA*^Ph−^ *LMNA* carrier of the pathogenic variant, *LMNA*^Ph+^ *LMNA* carrier phenotypically positive, *Med* median

### Diagnostic value of the validated circRNAs in a DCM population

The receiver operating characteristic (ROC) area under the curve (AUC) analysis was assessed to investigate the circulating circRNAs diagnostic value in discriminating *LMNA* and ischemic DCM etiology from healthy controls. All individual circRNAs show an AUC ≥ 0.7. The highest AUC values reached by hsa_circ_0089762 that demonstrated an AUC value of 0.92 (95% of confidence intervals [CI] range of specificities are shown in Table 3).

**Table 3:** Comparisons of single circRNA as predictors of DCM

DCM etiology	circRNA	AUC (95% CI)	Sensitivity (%)	Specificity (%)	*p*
*LMNA*	hsa_circ_0003258	0.75 (0.56–0.94)	61.53	78.57	0.043
	hsa_circ_0051238	0.71 (0.53–0.88)	70	72.73	0.02
	hsa_circ_0051239	0.73 (0.61–0.93)	83.33	72.23	0.007
Ischemic	hsa_circ_0089762	0.92 (0.77–1)	83.33	72.73	0.006

*AUC* area under the curve, *CI* confidence interval, *DCM* dilated cardiomyopathy, *LMNA lamin* A/C

### Association between the expression of circRNAs and the clinical characteristics of the DCM population

The association between circulating circRNAs and echocardiographic and clinical features of DCM patients was also analyzed. As indicated in Table 4, the *LMNA*^Ph−^ group showed a negative correlation between hsa_circ_0003258 and hsa_circ_0051239 with early diastolic mitral annular velocity (E’s TDI). The *LMNA*^Ph+^ cohort showed a positive correlation of hsa_circ_0051238 with tissue Doppler imaging (TDI) septal atrial systolic mitral annular velocity (A’s TDI) and a negative correlation of hsa_circ_0051238 and hsa_circ_0051239 with LV outflow tract (LVOT) velocity.

**Table 4:** Correlation between the echocardiographic variables and individual circRNA for the *LMNA* cohort

	**hsa_circ_0003258**						**hsa_circ_0051238**						**hsa_circ_0051239**					
	***LMNA***^**Ph−**^			***LMNA***^**Ph+**^			***LMNA***^**Ph−**^			***LMNA***^**Ph+**^			***LMNA***^**Ph−**^			***LMNA***^**Ph+**^		
	Pearson *r*	*p*	Power	Pearson *r*	*p*	Power	Pearson *r*	*p*	Power	Pearson *r*	*p*	Power	Pearson *r*	*p*	Power	Pearson *r*	*p*	Power
**A’s TDI (cm/s)**	0.191	0.623	0.677	0.676	0.324	0.584	− 0.013	0.965	0.966	0.859	**0.028**	0.928	0.051	0.875	0.882	0.698	0.190	0.554
**E’s TDI (cm/s)**	− 0.685	**0.042**	0.543	− 0.413	0.587	0.7	− 0.523	0.067	0.529	− 0.157	0.766	0.793	− 0.722	**0.008**	0.736	− 0.253	0.681	0.265
**LVOT (cm/s)**	− 0.085	0.856	0.87	− 0.995	0.064	0.927	− 0.020	0.963	0.8	− 0.977	**0.004**	0.965	0.043	0.919	0.564	− 0.97	**0.030**	0.923

*A’s TDI* atrial septal mitral annular velocity, *DCM* dilated cardiomyopathy, *E’s TDI* early diastolic mitral annular velocity, *LMNA lamin* A/C, *LMNA*^*Ph−*^* LMNA* carrier of the pathogenic variant, *LMNA*^*Ph*+^ *LMNA* carrier phenotypically positive, *LVOT* left ventricular outflow tract velocity, *TDI* tissue Doppler imaging

An additional study was performed to assess correlations between the echocardiographic and clinical variables and hsa_circ_0089762 for the ischemic DCM population. Hsa_circ_0089762 expression was negatively associated with diastolic blood pressure and LVEF (see Table 5).

**Table 5:** Correlation between the clinical parameters and hsa_circ_0089762 for ischemic DCM cohort

	**hsa_circ_0089762**		
	Pearson *r*	*p*	Power
**DBP (mm Hg)**	-0.84	0.036	0.556
**LVEF (%)**	-0.842	0.036	0.561

*DCM* dilated cardiomyopathy, *DBP* diastolic blood pressure, *LVEF* left ventricle ejection fraction

**Fig. 3:**
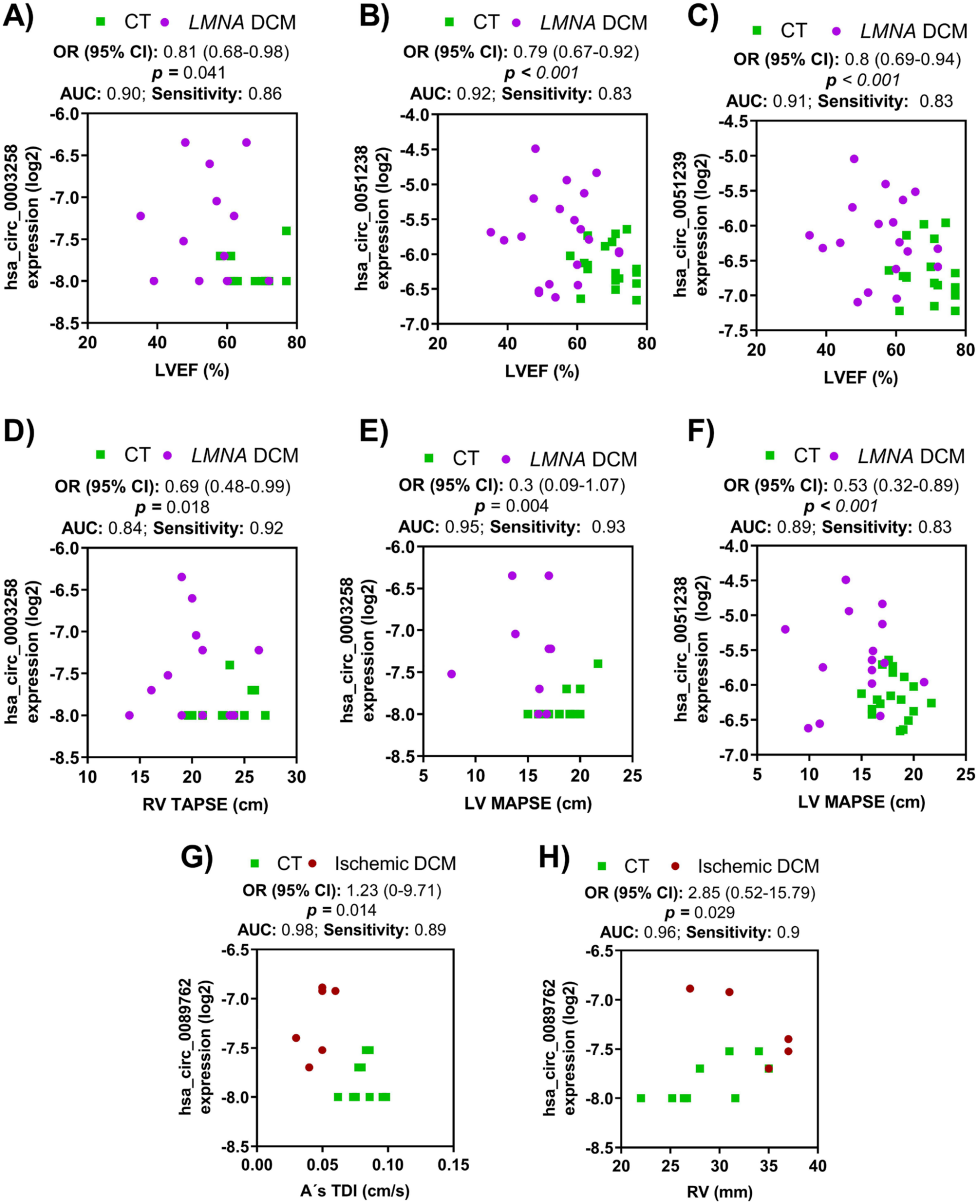
Bivariate logistic regression analysis for *LMNA*-related DCM and ischemic DCM patients. **A**–**F** Logistic regression analysis for the *LMNA*-related DCM cohort. LVEF was independently negatively related with hsa_circ_0003258 (**A**), hsa_circ_0051238 (**B**), and hsa_circ_0051239 (**C**). **D** RV tricuspid annular plane systolic excursion (TAPSE) was negatively related to hsa_circ_0003258. **E**, **F** LV mitral annular plane systolic excursion (MAPSE) was negatively correlated with hsa_circ_0003258 and hsa_circ_0051238. **G**, **H** The levels of hsa_circ_0089762 were associated with A’s TDI (**G**) and RV (**H**). The odds ratio, 95% of CI, and *p* values are indicated for each logistic regression analysis. Abbreviations: A’s TDI, atrial septal mitral annular velocity; AUC, area under the curve; CT, healthy group; CI, confidence intervals; *LMNA*, lamin A/C gene; LVEF, left ventricle ejection fraction; MAPSE, mitral annular plane systolic excursion; OR, odd ratio; RV; right ventricle; TAPSE, tricuspid annular plane systolic excursion

To further explore the expression of circRNA-DCM disease association, a logistic regression analysis was carried out in our DCM population (Fig. 3). All three *LMNA*-linked circRNAs were significantly related to male gender hsa_circ_0003258, hsa_circ_0051238, and hsa_circ_0051239. In the *LMNA* cohort, the bivariate logistic regression analyses revealed that all LVEF were independently negatively associated with hsa_circ_0003258, hsa_circ_0051238, and hsa_circ_0051239. LV mitral annular plane systolic excursion was independent negatively associated with hsa_circ_0003258 and hsa_circ_0051238. Right ventricle (RV) tricuspid annular plane systolic excursion was only independently negatively associated with hsa_circ_0003258. Pulmonary hypertension (PHT) was independently positively related to hsa_circ_0003258 and hsa_circ_0051239.

In the case of hsa_circ_0089762, the logistic regression analysis showed that its circulating levels within A’s TDI wave or the RV dimension were independent influencing factors for ischemic DCM.

### Annotation for circRNA/RBPs interaction

An examination of biological processes related to RBPs, with binding sites for circRNA candidates, reveals a set of possible pathways in which circRNAs play a regulative role. *LMNA* mutation influences the proper development of megakaryocytes resulting in altered platelet production/function [16]. We recovered this *LMNA* effect in the enrichment (GO:0,045,652), regulation of megakaryocyte differentiation (FDR = 0.0013), and fibroblast growth (GO:0,008,543) (FDR = 0.0267).

**Fig. 4:**
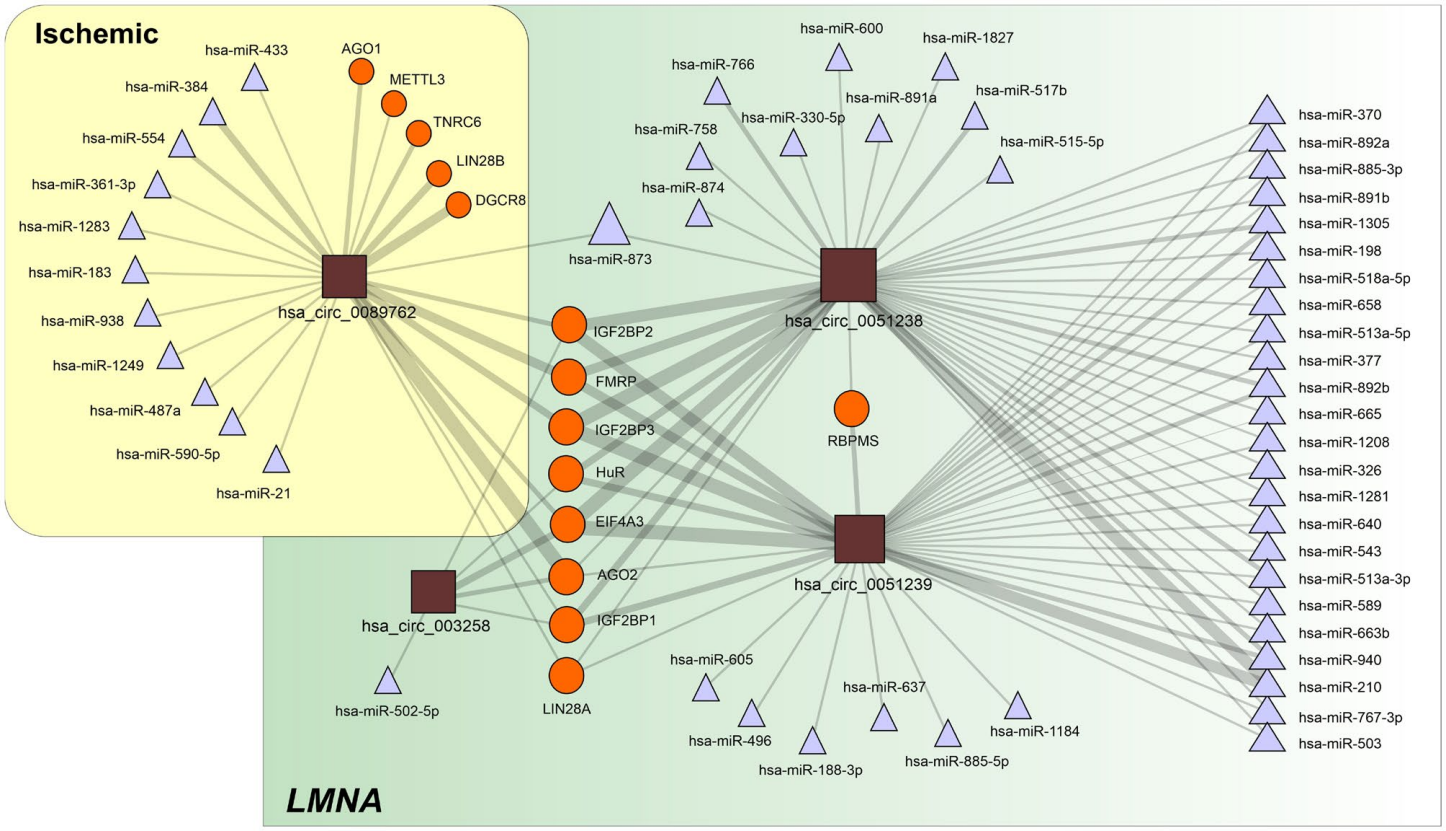
CircRNA-centered regulatory network established among the selected circRNAs. The depicted interactions are based on data extracted from the circInteractome database and include miRNAs and RBPs. CircRNAs are represented as squares, RBPs as circles and miRNAs as triangles. The size of each symbol is proportional to the number of interactions established. The edge thickness is also proportional to the number of targets for each interacting partner as included in the circInteractome database. The regulatory network was prepared with Navigator software [14]. Abbreviations: DCM, dilated cardiomyopathy; *LMNA*, lamin A/C gene; miRNA, microRNA; RBP, RNA-binding protein

The analysis of the intersection set of RBPs predicted to interact with the selected circRNAs (Fig. 4; Table 6) shows clear enrichment in proteins involved in the control of transcriptional and translational processes (Table 7). Note the association with the regulation of membrane potential, in which IEF4A3 and FMRP are involved (FDR = 0.045).

**Table 6:** Differentially expressed circRNA potentially interact with RBP and miRNAs

DCM etiology	circRNA	RBP	Predicted miRNAs target
*LMNA*	hsa_circ_003258	AGO2, EIF4A3, HuR, IGF2BP1, IGF2BP2	hsa-miR-502-5p
	hsa_circ_0051238	AGO2, EIF4A3, FMRP, HuR, IGF2BP1, IGF2BP2, IGF2BP3, LIN28A, RBPMS, ZC3H7B	hsa-miR-1208, hsa-miR-1281, hsa-miR-1305, hsa-miR-1827, hsa-miR-198, hsa-miR-210, hsa-miR-326, hsa-miR-330-5p, hsa-miR-370, hsa-miR-377, hsa-miR-503, hsa-miR-513a-3p, hsa-miR-513a-5p, hsa-miR-515-5p, hsa-miR-517b, hsa-miR-518a-5p, hsa-miR-543, hsa-miR-589, hsa-miR-600, hsa-miR-640, hsa-miR-658, hsa-miR-663b, hsa-miR-665, hsa-miR-758, hsa-miR-766, hsa-miR-767-3p, hsa-miR-873, hsa-miR-874, hsa-miR-885-3p, hsa-miR-891a, hsa-miR-891b, hsa-miR-892a, hsa-miR-892b, hsa-miR-940
	hsa_circ_0051239	AGO2, EIF4A3, FMRP, HuR, IGF2BP1, IGF2BP2, IGF2BP3, LIN28A, RBPMS	hsa-miR-1184, hsa-miR-1208, hsa-miR-1281, hsa-miR-1305, hsa-miR-188-3p, hsa-miR-198, hsa-miR-210, hsa-miR-326, hsa-miR-370, hsa-miR-377, hsa-miR-496, hsa-miR-503, hsa-miR-513a-3p, hsa-miR-513a-3p, hsa-miR-513a-5p, hsa-miR-518a-5p, hsa-miR-543, hsa-miR-589, hsa-miR-605, hsa-miR-637, hsa-miR-640, hsa-miR-658, hsa-miR-663b, hsa-miR-665, hsa-miR-767-3p, hsa-miR-885-3p, hsa-miR-885-5p, hsa-miR-891b, hsa-miR-892a, hsa-miR-892b, hsa-miR-940
Ischemic	hsa_circ_0089762	AGO1, AGO2, DGCR8, EIF4A3, FMRP, IGF2BP1, IGF2BP2, IGF2BP3, LIN28A, LIN28B, METTL3, TNRC6	hsa-miR-183, hsa-miR-21, hsa-miR-361-3p, hsa-miR-433, hsa-miR-590-5p

*DCM* dilated cardiomyopathy, *LMNA* lamin A/C, *RBP* RNA-binding protein

**Table 7:** Pathway analysis main findings: PPI enrichment analysis

Pathway	Overlap	FDR	Genes	Database
Regulation of translation	7/327	2.09e − 10	*AGO2,EIF4A3,ELAVL1,FMRP,IGF2BP1,IGF2BP2,IGF2BP3*	*GO*
mRNA binding	7/198	1.31e − 12	*AGO2,EIF4A3,ELAVL1,FMRP,IGF2BP1,IGF2BP2,IGF2BP3*	*GO*
Negative regulation of nitrogen compound metabolic process	7/2307	8.81e − 06	*AGO2,EIF4A3,ELAVL1,FMRP,IGF2BP1,IGF2BP2,IGF2BP3*	*GO*
Regulation of mRNA stability	5/113	1.05e − 08	*ELAVL1,FMRP,IGF2BP1,IGF2BP2,IGF2BP3*	*GO*
mRNA transport	5/148	3.12e − 08	*EIF4A3,FMRP,IGF2BP1,IGF2BP2,IGF2BP3*	*GO*
Regulation of gene silencing by miRNA	3/78	3.82e − 05	*AGO2,ELAVL1,FMRP*	*GO*
Regulation of membrane potential	2/408	0.0445	*EIF4A3,FMRP*	*GO*
MAPK6/MAPK4 signaling	2/86	0.0077	*AGO2,IGF2BP1*	*GO*
ncRNA processing	2/340	0.0344	*AGO2,EIF4A3*	*GO*

PPI enrichment *p*-value:1.67e − 10

*FDR* false discovery rate, *GO* gene ontology, *KW* keyword

The analysis of miRNAs sponged by validated circRNAs offers various candidates for further research. Hsa_circ_0003258 has only one functional binding site to hsa-miR-653. As a counterpart, hsa_circ_0051238 and hsa_circ_0051239 present a clear sponge effect over hsa-miR-210, with five binding sites that have ∆U below zero. Thereby, the overexpression of hsa_circ_0051238 and hsa_circ_0051239 will actively reduce the availability of hsa-miR-210. Hsa-miR-210 regulates expression of hepatocyte growth factor gene, whose overexpression is considered a treatment for DCM [17]. Additionally, they also present a functional binding site for hsa-miR-330-5p that is involved in cardiomyocyte survival and function recovery [18]. Regarding miRNA-related diseases, hsa_circ_0051238 sponges hsa-miR-873 and hsa-miR-513a-5p are both related with heart disease (*p* = 0.075), and hsa-miR-377 is related with ischemic cardiomyopathy (*p* = 0.221). Hsa_circ_0089762 has sponge activity with multiple, energetically favorable binding sites. Of note is hsa-miR-21, as well as hsa-miR-183, hsa-miR-361-3p, hsa-miR-384, hsa-miR-873, hsa-miR-938, hsa-miR-1249, and hsa-miR-1283. The miRNAs sponged by the circRNAs with a context score over 90% was used to capture the set of mRNAs regulated by these miRNAs. Functional enrichment, using a hypergeometric association algorithm, shows that 148 proteins of the network were related with focal adhesion (*p* = 2.68e^−8^), and 128 proteins were linked with regulation of the actin cytoskeleton (*p* = 0.00002). Gene ontology biological processes, using the same hypergeometric algorithm, show a significant correlation with endoplasmic reticulum-nuclei signaling pathways (*p* = 0.1e^−6^) and pre- and post-Golgi vesicle transportation (*p* = 8.6e^−7^ and 4.47e^−7^, respectively).

## Discussion

Over the last decade, the diagnostic process of DCM etiologies has focused on searching for new biomarkers. An efficient biomarker for DCM should be robust, stable, non-invasive, sensitive, specific to this entity, predictive of a particular DCM etiology, and show a preclinical and clinical relevance to be validated in animal and/or human cell models [19]. We propose the use of peripheral circRNAs as a novel discriminant biomarker of DCM etiologies.

Unlike linear RNA, single circulating circRNAs or circRNAs combined with various other biomarkers are a promising tool for clinical diagnosis of heart diseases, which would improve outcome [20]. Thus, circRNA MICRA was reported to risk-stratify patients after acute myocardial infarction [21]. Peripheral circ_0124644 and circ_0098964 levels have been described as a diagnostic biomarker of coronary artery disease [22]. Related to cardiomyopathies, a set of circulating circRNAs DNAJC6, TMEM56, and MBOAT2 has been proposed to discriminate between healthy and hypertrophic cardiomyopathy [23]. In this sense, hsa_circ_0071542 was upregulated in children with fulminant myocarditis in leukocytes isolated from peripheral blood [24]. Nevertheless, this area remains mostly unexplored in DCM [22, 25]. Recent studies have described several circRNA expression profiles in the DCM population compared to healthy patients. However, to date, it has not been studied among the different etiologies of DCM [26, 27]. Hence, further analysis of circRNAs among DCM etiologies might provide early, precise characterization of the disease and lead to novel pathological information, beyond the traditional biomarkers. To the best of our knowledge, the present study is the first to describe a subset of circulating circRNA for a discriminative etiology-based diagnostic in DCM. Circulating hsa_circ_0003258, hsa_circ_0051238, and hsa_circ_0051239 expression levels were upregulated in *LMNA*-related DCM patients. Notably, hsa_circ_0051238 plasmatic levels were significantly present in the *LMNA*^Ph−^ cohort. Hence, it may be a promising diagnostic biomarker for the early identification of patients in an initial stage of *LMNA*-related DCM. This will allow personalized therapeutic measures to be applied that help to improve the progression and outcome of *LMNA*-related DCM. Furthermore, plasmatic hsa_circ_0089762 may provide discriminative power for the ischemic DCM cohort with high-yield diagnostic accuracy and an AUC of 0.92. These circRNAs have been identified mostly in various types of oncologic processes [28–33]. Thus, only hsa_circ_0051239 levels have been upregulated in the myocardium of congenital ventricular septal defect [33]. However, they have not been previously described in DCM cases.

In the current study, circRNA were related to clinical and echocardiographic variables. Male gender, rare non-missense variants in *LMNA* and LVEF < 50% have been established as independent factors associated with a more aggressive outcome and even death during follow-up [34]. Herein, all three circRNAs associated with *LMNA-*DCM etiology were related to male gender [35]. On the other side, echocardiography variables and related circRNAs might suggest a time-evolving sequence. TDI echocardiography is a non-invasive, very sensitive method to assess the cardiac hemodynamic in DCM [36]. TDI reveals that subtle impairments in diastolic myocardial tissue velocities are markers of early cardiac disease and have been associated with outcome in various cardiopathies [16, 17]. In the *LMNA*^Ph−^ group, the E’s TDI is negatively related to hsa_circ_0003258 and hsa_circ_0051239. This E’s TDI impairment suggests an underlying early diastolic dysfunction [37]. A’s TDI in the *LMNA*^Ph+^ group showed a positive correlation, which indicates that the left atrium is a prominent factor to maintain the LV filling pressure when diastolic dysfunction advances. This sequential TDI septal impairment mirrors the transition from *LMNA*^Ph−^ to *LMNA*^Ph+^ and may be related to the progressive fibrosis of the interventricular septum located in the basal portion, which is characteristic of the *LMNA* related-DCM that has been associated with ventricular arrhythmias and worse prognosis [38]. LVEF was independently negatively associated with hsa_circ_0003258, hsa_circ_0051238, and hsa_circ_0051239. According to the LV systolic impairment, hsa_circ_0003258 and hsa_circ_0051238 were related to LV mitral annular plane systolic excursion. Thus, changes in contractility quantified by LV mitral annular plane systolic excursion occur as compensatory mechanisms before impairment of ventricular function [39]. Hsa_circ_0051238 and hsa_circ_0051239 were also negatively related to LVOT velocity, which suggests progressive impairment of the cardiac pump in the *LMNA*^Ph+^ cohort. Dysfunction of RV is a final common step in DCM and heart failure [40]. RV pressure overload due to PHT and the interventricular interdependence affected by septal fibrosis and underlying ischemia may influence this situation. In support of our results, circRNA, hsa_circ_0003258 was positively increased with the RV lower tricuspid annular plane systolic excursion and PHT [41].

Otherwise, hsa_circ_0089762 correlated to diastolic blood pressure and LVEF in the ischemic group, which supports our results as a specific, highly sensitive biomarker with high-yield diagnostic accuracy. Moreover, hsa_circ_0089762 was related to A’s TDI, which suggests more advanced progression of this entity. Its association with an increase in RV dimension could add information for tailored management in this group, since RV impairment is a worse outcome marker in the ischemic population [42]. In addition, RV involvement has a multifactorial origin that may be influenced by LV remodelling, increased LV filling pressures, and the appearance of PHT or RV ischemia [43].

Regarding biological implications, circRNAs spring from introns or exons of their parental genes by back-spliced circularization [25]. Hence, the ratio between linear and circular fractions affects gene expression. According to the protein atlas (proteinatlas.org), parental genes are expressed in cardiac tissue, which supports correlations between etiologies and circRNAs. Hsa_circ_0003258 is synthesized from *ZNF652* gene. ZNF652 interacts with CBFA2T3, which acts as a transcriptional repressor [44]. *ZNF652* is associated with systolic or diastolic blood pressure and hypertension. However, its role remains unclear [45]. Hsa_circ_0051238 and hsa_circ_0051239 come from the *ATP5SL* gene. *ATP5SL* is required for the assembly of mitochondrial NADH: ubiquinone oxidoreductase complex (complex I). Complex I is essential to provide the energy for cardiac function and is related to DCM progression [46]. *ATP5SL* has been associated with a congenital ventricular septal defect by the overexpression of hsa_circ_0051239 [47]. Finally, hsa_circ_0089762 is generated from the *MT-CO2* gene. MT-CO2 is part of the electron transport chain of the mitochondria. Reduced activity of the electron transport chain subunits has been described independently of etiology in ischemic or idiopathic DCM patients [48].

The functional enrichment of the intersecting set of RBSs reveals the role of FMRP in regulation of the membrane potential. Bao et al. described FMRP isoform 1, in rats, as an essential protection factor and a novel potential biomarker in the cardiovascular system [49]. The participation of circRNAs in regulatory networks involving competing-endogenous RNA interactions by sequestering miRNAs has been characterized recently in cardiovascular pathologies [50, 51]. From the set of miRNAs that could be sponged by the circRNAs that we considered, we found significant enrichment in the regulation of focal adhesion and actin cytoskeleton. Both have an important role in human DCM [52], which suggests new pathways of study.

Our current study has several limitations. Firstly, our sample was prospectively recruited from the outpatient clinic. The size of the study sample, comprised of strictly DCM patients, did not allow us to obtain a robust multivariate logistic regression model. Furthermore, a larger sample size is needed to validate these data by gender categorization since gender may play a role in the DCM prognosis [53, 54]. In consequence, these results should be extended and replicated to a larger population before the novel biomarkers can be routinely applied in clinical practice. Furthermore, data on natriuretic peptides or troponin were not accessible for all patients. Finally, even though databases registered the expression of the parental genes in cardiac tissue, we have no confirmation about the direct secretion from the heart of these circulating circRNAs into the extracellular space. Hence, the association of circRNAs with DCM and all the interactions are putative. Further analysis should be carried out on human heart samples to confirm our results.

## Conclusion

Exploring new biomarkers through circular transcriptome expression patterns will identify new targets in DCM pathogenesis. We propose a circulating circRNAs fingerprint to discriminate between various DCM etiologies. Circulating hsa_circ_0003258, hsa_circ_0051238, and hsa_circ_0051239 expression levels are higher in *LMNA*-related DCM, and hsa_circ_0089762 levels are specifically upregulated in the ischemic DCM cohort. These circulating circRNAs and certain echocardiographic variables might improve the etiology-based diagnostic, which allows early identification of asymptomatic cases and tailored treatment of the DCM population.

## Supplementary Information

Below is the link to the electronic supplementary material.

Supplementary file1 (PDF 456 KB)

## Data Availability

Data transparency is guaranteed. The datasets generated during and/or analyzed during the current study are available from the corresponding author on reasonable request.

## Code availability

We used various softwares for functional enrichment and statistical analysis. All of them are cited in our manuscript.

## Abbreviations

A’s TDI: Atrial septal mitral annular velocity
AUC: Area under the curve
*BAG3*: BCL2-associated athanogene 3
CI: Confidence interval
circRNA: Circular RNA
DCM: Dilated cardiomyopathy
E’s TDI: Early diastolic mitral annular velocity
FDR: False discovery rate
*LMNA*: Lamin A/C
LV: Left ventricular
LVEF: LV ejection fraction
LVOT: LV outflow tract
MAPSE: Mitral annular plane systolic excursion
mRNA: Messenger RNA
miRNA: MicroRNA
PHT: Pulmonary hypertension
ROC: Receiver operating characteristic
RBP: RNA binding protein
qRT-PCR: Quantitative real-time polymerase chain reaction
TAPSE: Tricuspid annular plane systolic excursion
TDI: Tissue Doppler imaging
