# Supplementary material for: Circulating circRNA as biomarkers for dilated cardiomyopathy etiology
Source: J Mol Med (Berl). 2021 Sep 8;99(12):1711–25. doi: 10.1007/s00109-021-02119-6 (PMC8599237; doi:10.1007/s00109-021-02119-6)
Supplement: Supplementary file 1 — Supplementary file1 (PDF 456 KB) [file 109_2021_2119_MOESM1_ESM.pdf]

**SUPPLEMENTARY INFORMATION**

# TITLE: CIRCULATING CIRCRNA AS BIOMARKERS FOR DILATED CARDIOMYOPATHY ETIOLOGY

Marina C. Costa [1], Maria Calderon-Dominguez [2*], Alipio Mangas [2,3,4], Oscar Campuzano[5,6,7], Georgia Sarquella-Brugada[5,6,7], Mónica Ramos[8], Maribel Quezada-Feijoo[8], José Manuel García Pinilla[9,10], Ainhoa Robles-Mezcua[9,10], Galan del Aguila Pacheco-Cruz[2],Thalia Belmonte[2], Francisco J Enguita[1,#], and Rocío Toro[2,4,#,*]

1. Instituto de Medicina Molecular João Lobo Antunes, Faculdade de Medicina, Universidade de Lisboa, Av. Prof. Egas Moniz 1649-028 Lisboa, Portugal; marinacosta@medicina.ulisboa.pt and fenguita@medicina.ulisboa.pt
2. Biomedical Research and Innovation Institute of Cadiz (INiBICA), Research Unit, Puerta del Mar University Hospital, Cádiz, Spain; mariacalderond@gmail.com, galapacheco@gmail.com, thaliabelmonte@gmail.com.
3. Internal Medicine Department, Puerta del Mar University Hospital, Cádiz, Spain; alipio.mangas@uca.es
4. Medicine Department, School of Medicine, University of Cádiz, Cádiz, Spain; rociotorogreen@gmail.com
5. Medical Science Department, School of Medicine, University of Girona (Spain); oscar@brugada.org, georgia@brugada.org
6. Cardiovascular Genetics Center, University of Girona-IDIBGI, Girona, Spain
7. Centro de Investigación en Red, Enfermedades Cardiovasculares (CIBEREV), Madrid, Spain.
8. Cardiology Department Hospital Cruz Roja, Alfonso X University, Madrid, Spain; monica.ramos81@gmail.com and maribelquezada2000@gmail.com
9. Servicio de Cardiología, Unidad de Insuficiencia Cardíaca y Cardiopatías Familiares, ,Hospital Universitario Virgen de la Victoria; IBIMA, Málaga, Spain; marlucale41@gmail.com and ainhoa.mezcua@gmail.com
10. CIBER-Cardiovascular, Instituto de Salud Carlos III, Ministerio de Sanidad, Consumo y Bienestar Social, Madrid,

* Correspondence: RT: rociotorogreen@gmail.com, MCD: mariacalderond@gmail.com,

**Supplemental figure 1**

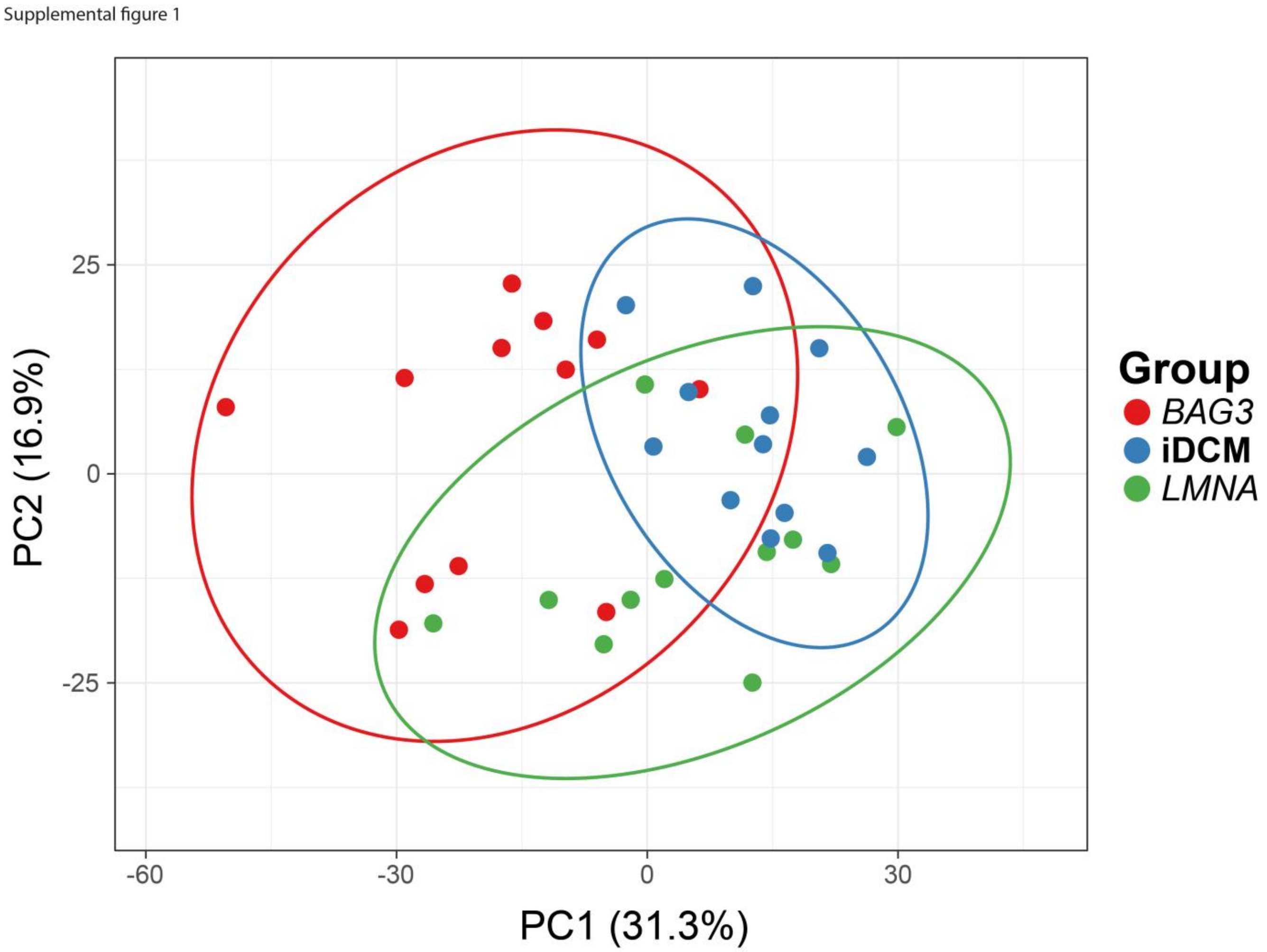

**Figure S1. The principle component analysis (PCA) on the microarray data.** PCA was showed on the differentially peripheral expressed circRNAs. Abbreviations: BAG3. BCL2-associated athanogene 3 DCM; DCM. dilated cardiomyopathy; iDCM; idiopathic DCM;  LMNA. lamin A/C DCM.

## Supplemental figure 2

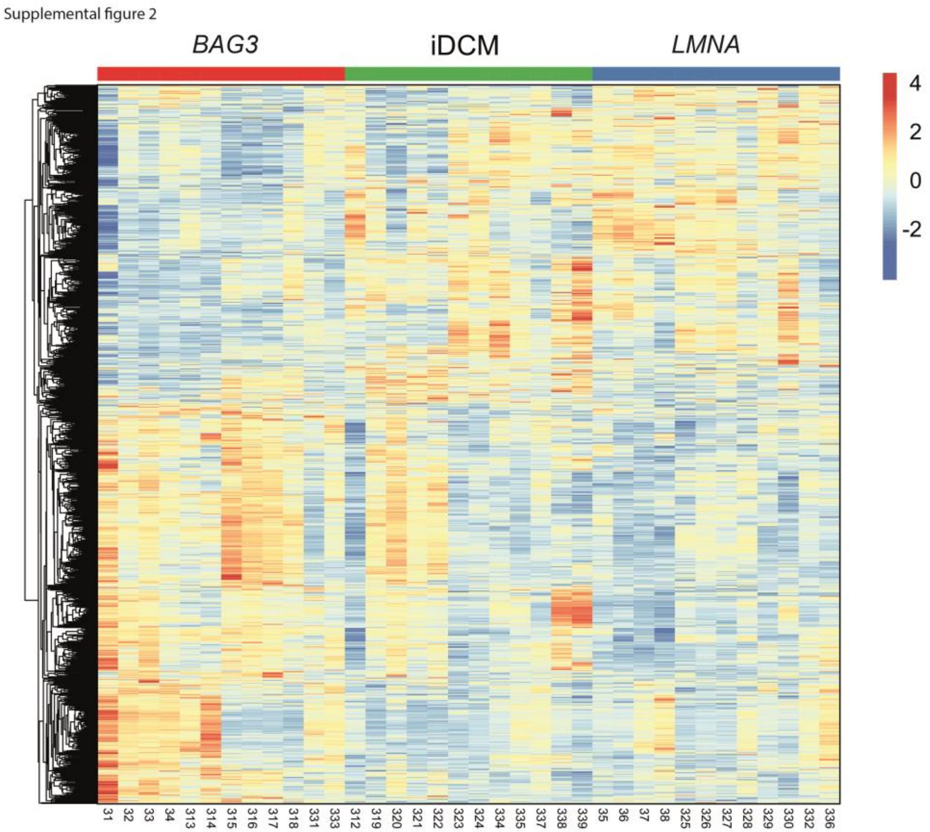


**Figure S2. Heatmap analyses of circRNA expression profiles.** Abbreviations: BAG3. BCL2-associated athanogene 3 DCM; DCM. dilated cardiomyopathy; iDCM; idiopathic DCM;  LMNA. lamin A/C DCM.

**Supplementary Table 1: primer sequences for circRNA quantification by qRT-PCR**

| circRNA (circBase) | Host gene | Forward primer | Reverse primer | Amplicon size |
|---|---|---|---|---|
| hsa_circ_0089762 | JA760602 | GGCGTGATCATGAAAGGTG | GGCCACCAATGGTACTGAAC | 177 |
| hsa_circ_0051239 | ATP5SL | ACACACACACACACGCACAC | AAGACCAAATCCCACATCCTC | 120 |
| hsa_circ_0051238 | ATP5SL | ACACACACACACACGCACAC | TTCTTCTGATTGCCCTCTGG | 128 |
| hsa_circ_0060144 | PHF20 | GAACCGACTTCTCCCCTTGT | CCCACTTCAAAGCTGATTCC | 128 |
| hsa_circ_0059760 | TM9SF4 | GCCATGTTCATCGAGCTCTT | CCTCCATGGTGTCTTCAATG | 120 |
| hsa_circ_0035957 | DENND4A | ACCACACACGTTCTGCAAAG | ACCGAAAGTTTCTCTCTGAAAAAG | 120 |
| hsa_circ_0003258 | ZNF652 | TGGGCACAAACAGTTCATGT | TGCGTTTGAATGATTTTCCA | 143 |
| hsa_circ_0089761 | JA760602 | GGCGTGATCATGAAAGGTG | CCCTAGCCAACCCCTTAAAC | 136 |
| hsa_circ_0023988 | NOX4 | CTGCTGACGTTGCATGTTTC | TCGGAGGTAAGCCAAGAGTG | 122 |
| hsa_circ_0089763 | JA760600 | TATGGTGGGCCATACGGTAG | CTCCACCTCCATCATCACCT | 136 |

**Supplementary Table 2. Peripheral circRNA levels in the study groups**

| circRNA | CT | | | *BAG3* DCM | | | | Idiopathic DCM | | | | Ischemic DCM | | | | *LMNA* DCM | | | |
|---|---|---|---|---|---|---|---|---|---|---|---|---|---|---|---|---|---|---|---|
| | Med | Q1 | Q3 | Med | Q1 | Q3 | *p* | Med | Q1 | Q3 | *p* | Med | Q1 | Q3 | *p* | Med | Q1 | Q3 | *p* |
| hsa_circ_ 0003258 | -8 | -8 | -7.92 | -8 | -8 | -7.03 | 0.38 | -7.55 | -8 | -7.04 | 0.23 | -8 | -8 | -7.52 | 0.89 | -7.61 | -8 | -6.93 | **0.02** |
| hsa_circ_ 0023988 | -5.99 | -7.76 | -3.59 | -8 | -8 | -7.39 | 0.37 | -6.76 | -8 | -5.53 | 0.9 | -7.69 | -7.69 | -4.76 | 0.87 | -7.76 | -8 | -7.52 | 0.49 |
| hsa_circ_ 0035957 | -7.52 | -8 | -7.16 | -7.46 | -8 | -7.12 | 0.65 | -7.39 | -8 | -7.30 | 0.98 | -7.52 | -7.52 | -7.09 | 0.60 | -7.61 | -7.77 | -7.22 | 0.97 |
| hsa_circ_ 0051238 | -6.20 | -6.39 | -5.9 | -6.21 | -6.39 | -5.94 | 0.92 | -5.90 | -6.25 | -5.33 | 0.15 | -5.87 | -6.30 | -5.51 | 0.21 | -5.78 | -6.43 | -5.27 | **0.04** |
| hsa_circ_ 0051239 | -6.73 | -6.92 | -6.48 | -6.85 | -7 | -6.26 | 0.91 | -6.33 | -6.79 | -5.88 | 0.07 | -6.33 | -6.87 | -5.89 | 0.27 | -6.24 | -6.59 | -5.70 | **0.01** |
| hsa_circ_ 0059760 | -8 | -8 | -7.77 | -8 | -8 | -8 | 0.9 | - | - | - | - | -7.52 | -7.52 | -7.52 | 0.06 | -8 | -8 | -8 | **0.77** |
| hsa_circ_ 0060144 | -8 | -8 | -7.76 | -8 | -8 | -7.04 | 0.85 | -8 | -8 | -7.69 | >0.9 | - | - | - | - | -8 | -8 | -7.28 | 0.85 |
| hsa_circ_ 0089761 | -7.22 | -8 | -6.72 | -8 | -8 | -7.32 | 0.25 | -7.84 | -8 | -7.39 | 0.23 | -8 | -8 | -7.30 | 0.23 | -8 | -8 | -7.69 | 0.08 |
| hsa_circ_ 0089762 | -8 | -8 | -7.52 | -7.69 | -8 | -7.39 | 0.49 | -7.84 | -8 | -7.40 | 0.67 | -7.15 | -7.57 | -6.91 | **0.04** | -7.52 | -8 | -7.15 | 0.27 |
| hsa_circ_ 0089763 | -8 | -8 | -7.52 | -7.84 | -8 | -7.53 | >0.9 9 | -8 | -8 | -7.46 | >0.9 | -7.69 | -8 | -7.46 | 0.99 | -7.84 | -8 | -7.26 | 0.99 |

Data presented as median (Q1-Q3). Coefficient significant at *p* <0.05. *BAG3*. BCL2-associated athanogene 3; CT. healthy control; DCM. dilated cardiomyopathy; *LMNA*. lamin A/C; $LMNA^{Ph-}$. LMNA carrier of the pathogenic variant; $LMNA^{Ph+}$. LMNA carrier phenotypically positive; Med. median.
